# Is foot ulcer simulation training (FUST) really effective? Participants’ supervisors speak out

**DOI:** 10.1186/1757-1146-6-S1-O24

**Published:** 2013-05-31

**Authors:** Vanessa Ng, Peter A Lazzarini, Patricia M Régo, Petrea Cornwell

**Affiliations:** 1 Allied Health Research Collaborative, Metro North Hospital & Health Service, Queensland Health, Brisbane, Queensland, 4032, Australia; 2 Department of Podiatry, Metro North Hospital & Health Service, Queensland Health, Brisbane, Queensland, 4032, Australia; 3 School of Clinical Sciences, Queensland University of Technology, Brisbane, Queensland, 4059, Australia; 4 School of Medicine, The University of Queensland, Brisbane, Queensland, 4072, Australia; 5 Clinical Skills Development Service, Centre for Healthcare Improvement, Queensland Health, Brisbane, Queensland, 4029, Australia; 6 Behavioural Basis of Health Program, Griffith Health Institute, Griffith University, Brisbane, Queensland, 4122, Australia

## Background

Foot ulcers are a common reason for diabetes-related hospitalisation. Foot ulcer simulation training (FUST) programs have increased podiatry participants self-confidence to manage foot ulcers. However, supervisors’ perspectives on their participants attending these simulation programs have not been investigated. This mixed method (quantitative and qualitative) study aimed to investigate home clinical supervisors’ perspectives on any changes to their participants’ competence and practice following FUST.

## Methods

Clinical supervisors of fifteen podiatrists, who participated in a two-day Foot Ulcer Simulation Training (FUST) course, were recruited. Supervisors completed quantitative surveys evaluating their participants’ foot ulcer competence pre-FUST and 6-months post-FUST, via a purposed designed 21-item survey using a five-point Likert scale (1=Very limited, 5=Highly competent). Supervisors also attended a semi-structured qualitative group interview to investigate supervisors’ perspectives on FUST.

## Results

Supervisors surveys returned were pre-FUST (n=10) and post-FUST (n=12). Significant competence improvements were observed at the 6-month survey (mean scores 2.84 cf. 3.72, *p* < 0.05). Five supervisors attended the group interview. Five sub-themes emerged: i) FUST provided a good foundation for future learning, ii) FUST modelled good clinical behaviour, iii) clinical practice improvement was evident in most participants, iv) clinical improvements were dependent on participant’s willingness to change and existing workplace culture, v) FUST needs to be reinforced back in the home clinic.

## Conclusion

Overall, supervisors of FUST participants indicated that the course improved their participants’ competence and clinical practice. However, the degree of improvement appears dependant on the participants’ home workplace culture and willingness to embrace change.

